# Comparison of long-term results of laparoscopic and endoscopic exploration of common bile duct

**DOI:** 10.4103/0972-9941.25672

**Published:** 2006-03

**Authors:** S S Rai, V V Grubnik, O L Kovalchuk, O V Grubnik

**Affiliations:** Department of Surgical Diseases and Post-Graduate Education, Odessa State Medical University, Odessa Regional Hospital, Street Zabalotnaya 26, Katovskawa, Odessa 65025, Ukraine - CIS

**Keywords:** Choledocholithiasis, ES, results, CBD stones

## Abstract

**Background:**

To compare long term results of laparoscopic and endoscopic exploration of common bile duct, to assess post-procedure quality of life.

**Materials and Methods:**

From September 1992 to August 2003, we performed 4058 cholecystectomies, out of which 479 (11.80%) patients had choledocholithiasis. There were 163 males and 316 females. Mean age was 63.65 ± 5.5 years. These patients were put in two groups. In the first group of 240 patients, a majority of patients underwent two-stage procedures. ERCP/ES was performed in 210 (87.50%) cases. In the second group of 239 patients, a majority of patients underwent single-stage procedures. ERCP/ES was done in 32 (13.38%) cases.

**Results:**

Mortality was zero in both groups. Morbidity was 15.1% in first group and 7.5% in second group. Mean hospital stay was 11.7 ± 3.2 days in first group and 6.2 ± 2.1 days in second group. Average operative time was 95.6 ± 20 minutes in first group and 128.4 ± 32 minutes in second group. Completed questionnaires received from 400 (83.50%) patients revealed better long-term results in the second group. Clinical features of low-grade cholangitis were seen in 20% of patients who underwent ES. Hence the post-procedure quality of life in patients who underwent single-stage procedures was definitely much better, because of minimal damage of sphincter of Oddi.

**Conclusions:**

Single-stage laparoscopic operations provide better results and shorter hospital stay. Damage to sphincter of Oddi should be minimal, to avoid long-term low-grade cholangitis. In young patients, the operation of choice should be single-stage laparoscopic procedure with absolutely no damage to sphincter of Oddi.

The management of common bile duct stones (CBDS) remains controversial. There is no standard algorithm and the disparity in laparoscopic skills among surgeons has perpetuated this lack of a standard.[1] This situation is reflected in practices and studies that describe either a complete reliance on endoscopic retrograde cholangiopancreatography (ERCP), or persistent common practice of open common bile duct exploration (CBDE) for management of choledocholithiasis.[2,3] The current options available for the management of choledocholithisis at the time of LC, include pre-operative ERCP and ES, intra-operative ERCP, post-operative ERCP, laparoscopic transcystic common bile duct exploration (LTCCBDE), laparoscopic choledochotomy and LCBDE and open bile duct exploration. In our clinic, we use a balanced approach to the management of the choledocholithiasis.

## MATERIALS AND METHODS

From September 1992 to August 2003, 4058 LC were performed in our clinic. 2678 (65.99%) patients were operated for chronic cholecystitis and 1380 (34%) for acute cholecystitis. There were 2962 (72.99%) women and 1086 (26.76%) men. The mean age of the patients was 54.8 years (range 5–84 years). 29% of patients were classified as high-risk ASA III-IV. Pre-operative examination was done through medical history, biochemical tests and ultrasonography in all patients. 10% patients had CT and MRI examination before operation.

During the period 1992 through 1997, in the patients with clinical features of choledocholithiasis, the first step was ERCP. If ERCP confirmed CBDS, we performed endoscopic sphincterotomy (ES). In the cases of suspected CBDS during LC, the diameter of CBD was measured with a special instrument. If it was > 8 mm and cystic duct was > 4 mm, intra-operative cholangiography (IOC) was indicated. LTCCBDE was attempted, if CBDS were confirmed.

During the second phase from 1997 to 2003, most patients were treated by laparoscopic procedures. If there was a high suspicion of choledocholithiasis in a patient with significant co-morbidities, a pre-operative ERCP and ES was performed followed by LC. Otherwise, LC was done straight-away. In the cases of choledocholithiasis, we tried to perform LTCCBDE or choledochotomy and LCBDE. Post-operative ERCP was used, if these methods of treatment of choledocholithiasis were incomplete. Open CBDE was reserved for those cases that failed to respond to the minimally invasive maneuvers.

### Technique

ERCP was performed by standard techniques. Laparoscopic IOC was performed by inserting a standard cholangio-catheter into a nick in cystic duct. LTCCBDE was performed after initially evaluating the characteristics of cystic duct, CBD and stones. This procedure was often facilitated by balloon dilatation of cystic duct, and augmented by use of wire baskets, balloon catheters, choledochoscopy and electrohydraulic lithotripsy (EHL). For a few relatively small stones, a spiral basket was passed fluoroscopically for stone retrieval. If these measures failed clearing the duct, choledochoscopy was performed. We utilized a 3.4 mm outer diameter choledochoscope with a 3.6 Fr inner channel (Karl Storz Endoscopy), or used a 2.7 mm outer diameter Olympus choledochoscope. The choledochoscope was used to guide basket retrieval, or to assess the success of other maneuvers. For large or impacted stones, EHL through choledochoscope was used for fragmentation. The particles were then either retrieved transcystically, or flushed through the ampulla. If the anatomy was unfavorable for transcystic approach, or if there were too many stones (>5) and CBD was of sufficient size, a choledochotomy and LCBDE were performed. If there were stones 5–7 mm in diameter and stenosis of papilla, an intra-operative ES and balloon stone extractions were performed. In some patients, we performed post-operative ERCP and ES for clearing CBD. In presence of cholangitis, we used a T-tube or transcystic drainage. In other cases, we closed choledochotomy using a biliary stent. The patients were followed up in the out-patient clinic at a 3–6 months interval.

## RESULTS

The chi-square test and Fisher's exact test were used for analysis of data. Statistical significance was set at *P*<0.05. Between September 1992 and August 2003, we performed 4058 LC. Stones in CBD were detected in 479 (11.8%) cases, 163 (34.02%) patients were males and 316 (65.97%) were females; their mean age was 61.65 ± 4.85 years.

In the first group 240 patients with choledocholithiasis were operated. There were 78 males and 162 females. Mean age of the patients was 62.8 ± 4.5 years. ERCP before LC was performed in 228 patients In 210 (92.10%) patients, ERCP was performed successfully, CBDS were detected in 202 (96.19%) patients and all of them underwent ES. ERCP failed in the remaining 18 patients due to changed anatomy after Billroth II resection and a duodenal diverticulum. Amongst these 18 patients, 10 underwent CBDE (open in 8 and laparoscopic in 2). The symptoms of the other 8 disappeared spontaneously.

ES was attempted in 202 patients and succeeded in 182 (90.09%) patients. 32 patients had large stones, which were crushed and a Fogarty catheter was used to wash them out. In 5 patients, it was impossible to retrieve the large stones. Of the 9 patients in whom no duct clearance was obtained, 4 underwent open cholecystectomy with CBDE and 5 patients had successful LCBDE.

ERCP/ES-related complications occurred in 20 (10.9%) patients. In 10 (5.5%) patients, bleeding occurred, necessitating a laparotomy in 2 patients and prolonged hospital stay with transfusion in 12 patients. 6 (3.3%) patients developed post-procedure pancreatitis. Cholangitis and sepsis were seen in 3 patients (1.6%) and these patients underwent laparotomy. Open CBDE with T-tube drains was performed. 1 patient suffered duodenal perforation and this patient required laparotomy.

Amongst patients in whom ERCP/ES was performed before LC, there were some further complications; raising the number to 25 (13.73%) patients; in 20 patients after ES and in 5 patients during LC. Conversion to open procedure was done in 8 patients due to technical difficulties, Mirizzi syndrome and incomplete clearance of CBD.

IOC during LC in 40 patients in the first group detected CBDS. LTCCBDE was successfully performed in 12 patients. The remaining 28 patients underwent post-operative ES, with clearance of CBD. Complications occurred in 4 patients in whom ES was performed after LC. Acute pancreatitis: 2, bleeding: 1 and duodenal perforation: 1 (this patient underwent laparotomy).

In the first group, two-stage laparoscopic and endoscopic procedures were performed in 210 (85.7%) patients. One-stage operation; LC + LTCCBDE was performed successfully only in 12 (5%) patients. Number of open operations was 18 (7.5%) in the patients. Mortality was 0 and morbidity was 13.3%. Mean hospital stay was 11.7 ± 3.2 days [Table 1].

**Table 1:** Comparison of results of treatment of the two groups of the patients with choledocholithiasis

Indices	Group I 1992–1997	Group II 1998–2003
Number of patients	N=240	N=239
Sex - Male/Female	78/162	85/154
Mean age	62.8 ± 4.5 years	60.5 ± 5.2 years
Pre-operative ERCP/ES	182	14
LC + LTCCBDE	12	119
LC + LCBDE	-	68
LC + intra-operative ES	-	14
LC + post-operative ERCP/ES	28	18
Open operations	18	6
Conversion rate	4.2%	2%
Morbidity	32 (13.3%)	18 (7.5%)
Mortality	0	0
Mean hospital stay	11.7 ± 3.2 days	6.2 ± 2.1 days
Average operative time	95.6 ± 20 mins	128.4 ± 32 mins

During the second period, 239 patients with choledocholithiasis were operated. There were 85 (35.56%) males and 154 (64.43%) females. Mean age was 60.5 ± 5.2 years. Pre-operative ERCP with ES was performed in 14 (5.85%) patients. Failure of stone extraction occurred in 4 patients. Most of these failures were attributable to multiple, large and impacted stone during ERCP. LCBDE was performed in these patients.

ERCP/ES-related complications were seen in 2 patients (bleeding: 1, acute pancreatitis: 1). Most of the patients in second group were operated laparoscopically. LTCCBDE was all that was required to completely clear the CBD in 119 cases (71.68% success rate). Reasons of failure of LTCCBDE were: too many stones (or stone-fragments after EHL): 12, stones in proximal biliary ducts: 6, inability to dilate the cystic duct: 5, too large stones in CBD: 12. 14 patients underwent intra-operative ERCP without CBDE, because of significant inflammation around the cystic duct: 4, multiple stones and stenosis of papilla: 10. The average operative time for these patients was 138 minutes (range 118–192 minutes). Overall, 14 patients had an intra-operative ERCP/ES, acute pancreatitis was developed in 1 patient.

Complications after LTCCBDE were seen in 6 (5%) of 119 cases. Bile leakage occurred in 2 patients and they were re-operated. In one patient, re-laparoscopy was done in another post-operative ES. In 4 patients, minor complications were successfully managed by puncture and drainage. In long-term follow-up (32 months), residual stones were detected in 3 patients from this group. They underwent ES with good results.

Laparoscopic choledochotomy was executed in 68 patients. The mean duration for combined LC and laparoscopic choledochotomy was 128 min (range 72–186 minutes). Complete retrieval of stones via laparoscopic choledochotomy was achieved in 64 (94.11%) patients. At the end of exploration, biliary stents were inserted in 28 (41.17%) patients. T-tubes or drains through cystic duct were placed in 36 (52.94%) patients. Primary closure of choledochotomy incision using antegrade biliary stents were done in 32 (47%) cases.

There were 3 conversions, due to injury to rear wall of CBD. 3 patients experienced bile leakage at the choledochotomy site, which resolved with conservative management in one patient. Two patients were re-operated (one laparotomy and one re-laparoscopy) for treatment of bacterial peritonitis and bile leakage. In 2 patients, sub-diaphragmatic abscess and biloma were punctured under ultrasound. Morbidity rate in the patients who had laparoscopic choledochotomy was 8.82% (6 cases). Post-operative ERCP/ES were performed in 18 (7.53%) patients in the second group. Complications were observed in 3; bleeding: 2, severe pancreatitis: 1. The last patient required laparotomy.

The mortality rate was zero among all patients with CBDS. Morbidity rate was higher in the first group of the patients [Table 1] (*P*<0.05). Mean hospital stay was 11.7 ± 3.2 days in the first group of patients and 6.2 ± 2.1 days in the second group (*P*<0.05). In the group of patients who underwent laparoscopic choledochotomy, mean hospital stay was 8.4 ± 3.2 days, compared with 4.6 ± 2.5 days in patients who underwent transcystic exploration. The average operative time was 95.6 ± 20 mins in the first group, whereas it was 128.4 ± 32 mins in the second group (*P*>0.05).

Careful analysis of [Table 1] reveals different surgical operative options providing different results. Number of complications in the first group in which two-staged procedures were performed was higher (*P*<0.05). It was so because of adding up of complications of ES and LC. Mean hospital stay was also longer in the first group (*P*<0.05), this is explained by the extended treatment in case of occurring of complications. Operative time in the second group was slightly higher, because one-stage procedures were executed comprising of LC and LCBDE (*P* >0.05), even though it did not have any substantial influence on the number of complications and the quality of treatment.

In the second group, among 20% of patients, it was necessary to perform ES for complete extraction of stones. ES was executed in combination with LC in 210 patients in the first group and in 46 patients in the second group. From these 256 patients, long-term results ranging from 3 to 8 years were studied in 207 (81.85%) patients. 14 patients died after operation. In 7 patients, the cause of death was related to cholangitis, sepsis and hepatic insufficiency and also complications from the repeated operations. From 187 patients in which LCBDE was performed without ES, long-term results ranging from 1 to 6 years were studied in 169 (90.37%) patients. 2 patients died from reasons unconnected with operation and pathology of biliary ducts.

For long-term follow-up, letters with the questionnaires were sent to all the patients every 6 months. Patients were asked to indicate whether they experienced a variety of symptoms compatible with low grade cholangitis. In addition, they were asked to give details of any further endoscopic or open treatment for biliary disorders they may have undergone, as well as any admissions to hospital. Finally, they were asked to indicate whether they ever suffered ‘off days,’ and whether they considered their general health worse, the same or better.

Completed questionnaires were received from 207 patients from the first group (response rate: 86.25%). 193 patients who are now alive were inspected in our clinic and good results were registered in 157 (81.34%) patients. Symptoms of low-grade cholangitis were detected in 20.36% patients [Table 2], residual stones in bile ducts were detected in 9 (4.66%) patients, re-stenosis of papilla was observed in 10 (5.18%) patients and chronic pancreatitis was seen in 12 (6.21%) patients. From the second group, completed questionnaires were received from 187 patients (response rate: 78.24%). In these patients, LCBDE was done without ES. 167 patients who are now alive were inspected in our clinic and good results were registered in 152 (91.01%) patients. Recurrent cholangitis was detected only in 6 (3.94%) patients. 2 (1.31%) patients revealed residual stones and in 2 (1.31%) patients, recurrent stones were detected. In all of these patients, ES was carried out and good results were obtained.

**Table 2:** Patients reporting symptoms attributable to recurrent sub-clinical cholangitis in patients who underwent ES (A) and patients who did not undergo ES (B)

Symptoms	Occasionally		Often	
		
	A (%)	B (%)	A (%)	B (%)
Nausea	52 (26.9)	12 (7.2)	13 (6.7)	5 (3)
Vomitin	35 (18.1)	6 (3.6)	10 (5.2)	2 (1.2)
Jaundice	14 (7.2)	4 (2.4)	9 (4.7)	2 (1.2)
Rigors	35 (18.1)	6 (3.6)	5 (2.6)	2 (1.2)
Dark urine	32 (16.6)	8 (4.5)	9 (4.7)	4 (2.4)
Pale stools	48 (24.9)	10 (6)	10 (5.2)	4 (2.4)
Fever	18 (9.3)	10 (6)	8 (4.1)	6 (3.6)
Sweats	37 (19.2)	-	14 (7.3)	-

10 patients were re-investigated with an ERCP and ES for episodic jaundice and re-stenosis of papilla. 2 patients were re-operated, open CBDE was performed.

A careful analysis of table 2 reveals that the frequency of symptoms associated with low grade cholangitis were less in the second group (*P*<0.05), the explanation is less damage to the sphincter of Oddi.

## DISCUSSION

One stage operations have some benefits, as compared to two stage operations. Morbidity after one-stage operations was only 7.5% (2 times lower), in our series. The reported results of LCBDE when compared to data obtained after the two-stage procedure, show at least identical, rather improved safety for the patient and partial reduction of costs.[4–10] Cuschieri *et al*[11] reported a prospective randomized multi-center trial, showing similar success and complication rates and a significant reduction of hospital stay for single stage management of choledocholithiasis. Furthermore, it is shown that particularly patients with ASA stage I and II benefit from simultaneous laparoscopic surgery and that high-risk patients should undergo primary ES. According to a recent publication, LCBDE can also be conducted safely with a low complication rate in older patients.[12] We had a similar experience with our therapeutic concept. Obtaining definitive stone retrieval (after transcystic approach, success was obtained in 72% after laparoscopic choledochotomy, successful retrieval of stones was 94%). We have a complication rate of 7.5%, which interestingly is two times lower than after two-stage operations. Our data is comparable with data reported in literature.[1,4,6,9,11,13] Advantageous impact on shorter hospital stay was observed in our series. Hospital stay was 12.7 ± 3.8 days after two-stage operations and 7.2 ± 2.1 days after a one-stage operation. We prefer a single-stage procedure.

Although some authors have suggested primary closure of the choledochotomy without drainage,[14] we believe that for the safety of the patient, bile duct decompression must be achieved. Despite of its advantages, T-tube has significant complications such as post-operative bacteremia, stone formation around the tube, skin excoriations at the exit site, prolonged biliary fistula, retention of a fragment of the tube, late bile duct stricture and dislodgement of the tube with subsequent bile peritonitis and sepsis leading to mortality.[11,15,16] Moreover, these complications seem to be unrelated to the techniques used.[2,17–19]

All forms of external biliary drainage should be avoided in the ideal single stage procedure for sake of the patient's comfort. Although the use of modified biliary stent obviates the discomfort and complications associated with an external drainage tube, it also has some disadvantages. With this approach, cholangiogram is not feasible, no fistulous tract is available for removal of retained stones and endoscopic removal of stent is sometimes necessary. In our option, the benefits of the stent out weigh the disadvantages.

Controversy still exists concerning impaired function of the papilla following ES. Soehendra *et al*,[3] reports absence of papilla function impairment. In contrast, other groups report reflux of duodenal secretion into bile ducts and presence of bacteria in the biliary ductal system in 70% of cases[20–22] and significant biliary symptomatology in 15% of patients.[17,23,24] Tranter and Thompson reported a late development of bile duct cancer in upto 2% of patients following ES, possibly based on the chronic mucosal inflammation.[25] In contrast, LCBDE provides the anatomical and functional integrity of the papilla.

Clearly there is no single best approach for the management of choledocholithiasis. The optimal treatment is one that can be performed in the same setting as LC, while maintaining a minimal invasive approach. LTCCBDE meets these requirements with the lowest rate of morbidity, but admittedly it is not always possible, or successful. Laparoscopic choledochotomy has proven to be safe and successful, but it is appropriate only when less invasive means have failed or are expected to fail and the duct is of appropriate size. ERCP/ES is useful as a primary treatment in the group of patients with jaundice and severe co-morbidities. ERCP/ES can also be a good option for the post-operative management of retained stones, but it should not be relied upon routinely in lieu of intra-operative management. Most cases of choledocholithiasis can be managed at the time of LC. This approach decreases the patient's hospital stay and the overall cost of treatment.

## CONCLUSIONS

1. While selecting the operative technique to treat choledocholithiasis, single-stage procedures should be given the priority, as they cause less morbidity and short hospital stay.
2. Damage to sphincter of Oddi should be minimal to obtain better long-term results.
3. Consideration of post-procedure quality of life should be given high priority.
4. In young patients, laparoscopic single-stage procedures should be used to extract stones and the sphincter of Oddi should not be cut.
